# Dynamic remodeling of skin microbiota during healthy homeostatic and wound repair conditions

**DOI:** 10.3389/fmicb.2026.1781606

**Published:** 2026-02-19

**Authors:** Hailv Ye, Aloysius Wong, Xiong Chen, Wenxuan Sun, Huacheng He, Xiaokun Li, Jiang Wu

**Affiliations:** 1 Department of Biology, College of Science, Mathematics and Technology, Wenzhou-Kean University, Wenzhou, Zhejiang, China; 2 School of Pharmaceutical Sciences, Key Laboratory of Biotechnology and Pharmaceutical Engineering, Wenzhou Medical University, Wenzhou, Zhejiang, China; 3 The International Frontier Interdisciplinary Research Institute, Wenzhou-Kean University, Wenzhou, Zhejiang, China; 4 Department of Wound Repair, The First Affiliated Hospital of Wenzhou Medical University, Wenzhou, China; 5 Oujiang Laboratory, Zhejiang Lab for Regenerative Medicine, Vision and Brain Health, Wenzhou, Zhejiang, China

**Keywords:** commensal–host interactions, cutaneous immunity, microbiome-based therapies, skin microbiome, wound healing

## Abstract

The skin microbiota is a fundamental component of the cutaneous ecosystem and plays an important role in maintaining skin homeostasis through immune education, maintenance of the skin barrier, colonization resistance, and regulation of the physiological environment under healthy conditions. Skin injury disrupts this balanced microbial ecosystem, resulting in marked changes in the local microenvironment. However, the processes by which skin microbiota reorganise following injury and contribute to the restoration of a remodelled homeostatic ecosystem after wound repair are not fully understood. This review synthesizes current knowledge on host–microbiota interactions across the dynamic transition from healthy skin to wounded skin and to remodeled homeostatic skin. We highlight the functions of commensal microorganisms during the inflammatory, proliferative, and remodeling phases of wound healing, with a particular focus on their roles in the resolution of inflammation, tissue regeneration and barrier restoration. Finally, we discuss emerging microbiota-based therapeutic opportunities for wound management and outline key challenges and future research directions aimed at promoting long-term restoration of skin microbial homeostasis.

## 1 Introduction

The skin is the largest organ of the human body. It functions as a physiological barrier against external pathogens and as a habitat for diverse and complex microbial communities (Peate, 2021). The skin microbiota plays a crucial role in sensing environmental stimuli, regulating host immune responses, and maintaining cutaneous homeostasis. Based on their functional characteristics, resident microbes are broadly divided into commensals and potential pathogens (Cogen et al., 2008). Studies have shown that commensal species such as *Staphylococcus epidermidis* (*S. epidermidis*), *Cutibacterium acnes* (*C. acnes*), and *Corynebacterium* spp. are closely associated with immune regulation and barrier maintenance (Rozas et al., 2021; Zheng et al., 2022; Bay and Ring, 2022), whereas other species such as *Staphylococcus aureus* (*S. aureus*), *Streptococcus* spp., *Escherichia coli*, and *Candida albicans* are commonly are commonly detected in inflamed or diseased skin (Chiller et al., 2001). The microbial composition of healthy human skin differs from that of wounded skin. In healthy skin, the most prevalent bacterial genera include *Cutibacterium* (approximately 23%), *Corynebacterium* (approximately 22.8%), and *Staphylococcus* (approximately 16.8%) (Grice et al., 2009). In contrast, wounded skin is typically dominated by *Staphylococcus*, *Pseudomonas*, *Corynebacterium*, *Streptococcus*, *anaerobic cocci*, and *Enterococcus* (Purohit and Solanki, 2013).

Under healthy conditions, microbial communities on the skin maintain a highly dynamic and finely tuned equilibrium. This microecological homeostasis is crucial for maintaining barrier function, preventing pathogen invasion, and regulating basal inflammatory responses. Through continuous interactions with keratinocytes, innate immune cells, and adaptive immune cells, the microbiota collectively shapes cutaneous immune stability. When this ecological balance is disrupted, immune dysregulation and heightened inflammation may occur, increasing susceptibility to infection and impairing tissue function (Bay and Ring, 2022). Among these conditions, the wound environment represents the most pronounced and rapidly evolving form of microecological imbalance. Following injury, the initial wound microenvironment is profoundly disrupted. While some wounds successfully transition toward resolution through microbiota-mediated regulation, others remain trapped in a prolonged state of dysregulation, leading to chronic non-healing wounds. Impaired wound healing has long been attributed to microbial virulence factors and biofilm formation, both of which can sustain inflammation and hinder tissue repair (Metcalf and Bowler, 2013; Percival et al., 2012; Kalan et al., 2019). The beneficial roles of skin commensals during the inflammatory, proliferative, and remodeling phases are particularly critical. Wound healing should therefore not be viewed as a static condition of sustained dysbiosis, but rather as a dynamic process of microecological reorganization and restoration. Growing evidence indicates that under specific immune and environmental conditions, certain microbes can promote repair (Di Domizio et al., 2020; Constantinides et al., 2019; White et al., 2024; Harrison et al., 2019). In these review, microorganisms may transition from passive colonizers to functional modulators that participate in inflammation resolution, re-epithelialization, and tissue reconstruction (Gan et al., 2024). These findings suggest that the dynamics of the wound microbiome are more complex than previously recognized, and their underlying mechanisms and regulatory potential require further investigation.

These observations prompt an important question: can we harness the beneficial functions of skin commensals to restore the wound microenvironment and promote tissue repair? The answer depends largely on how the skin immune system senses and interprets microbial signals. We synthesize current evidence on host–microbiota interactions across skin homeostasis, wound repair, and post-healing stages, with a particular focus on the positive roles of commensal microbes. This perspective further highlights the potential of leveraging skin microbes or their metabolites as therapeutic agents to modulate the wound microenvironment and facilitate tissue repair.

## 2 Skin microbiota dynamics under healthy homeostatic conditions

In healthy individuals, a balanced skin microbiome supports immune education, barrier maintenance, and colonization resistance. Early-life colonization by commensals is essential for immune development, while insufficient microbial exposure may lead to dysregulated responses later in life. Notably, studies have shown that wound healing is delayed under germ-free conditions, underscoring the supportive role of commensal microbes (Wang et al., 2021). In addition, decreased microbial diversity such as that observed in diabetic skin without visible wounds has been linked to impaired healing (Bay and Ring, 2022). Thus, healthy skin microbiota profiles under homeostatic conditions may serve as predictors of wound healing potential.

### 2.1 Maintenance of a stable immune system by microbiome in healthy skin

**Figure 1:**
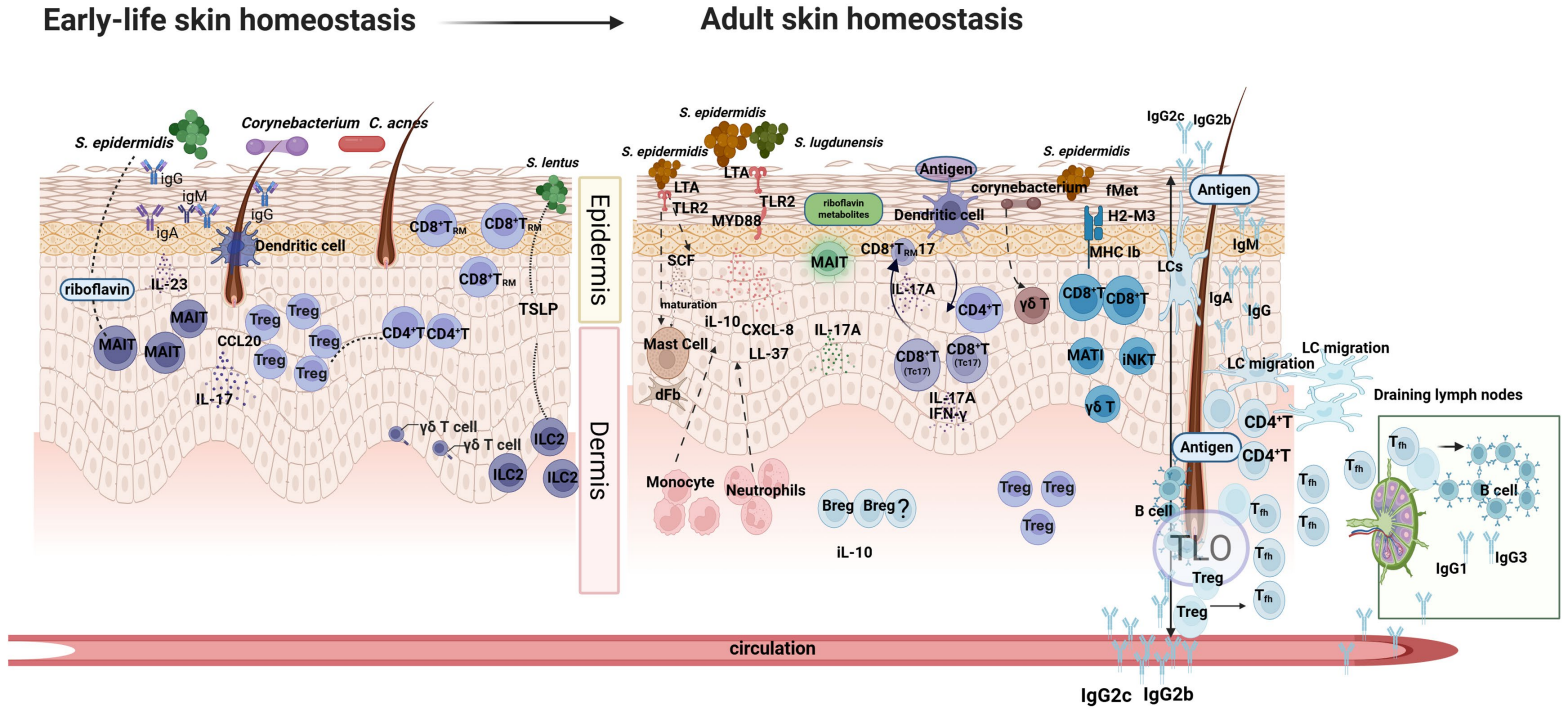
Early-life versus adult skin microbial colonization and associated immune responses. Early-life exposure to commensals promotes the establishment of key immune populations, including Tregs, CD4^+^, and CD8^+^ T cells, MAIT cells, γδ T cells, and ILCs, and supports initial antibody production by innate-like B-1-like cells, thereby shaping foundational cutaneous immune homeostasis. In adulthood, microbial components activate keratinocytes and innate immune cells through TLR2–MyD88 signaling, inducing the production of antimicrobial peptides, CXCL8, and IL-10; additionally, LTA promotes mast cell maturation. DC presentation of commensal antigens drives the activation of Th17, Tc17, and T_RM_17 cells, which produce IL-17A to reinforce epithelial barrier function. MHC Ib further activate cells, contributing to CD8^+^T, MATI, iNKT and γδ T. Skin B cells produce IgA, IgG, and IgM detected on the skin surface, coating microbes and strengthening humoral defense. TLOs can form in healthy skin, where *S. epidermidis*–induced Tfh responses promote local antibody production and support long-lived plasma cells and memory B cells. Bregs contribute to immune tolerance by limiting excessive activation against commensals. Created with BioRender.com. *TSLP* Thymic Stromal Lymphopoietin, *MAIT* Mucosal-Associated Invariant T cells, *Treg* Regulatory T cells, *ILC2* Group 2 Innate Lymphoid Cells, *Breg* Regulatory B cell, *TLO* Tertiary lymphoid organ, *LC* Langerhans cell, *TLR* Toll-like receptor, *LTA* Lipoteichoic acid, *fMet* N-formyl methionine–containing peptides, *DC* Dendritic cell, NKT natural killer T.

The epidermis and dermis together form a coordinated network of immune and non-immune cells that maintains barrier integrity, supports epithelial renewal, and protects against pathogen invasion. As an active barrier, the skin microbiota dynamically interacts with keratinocytes and multiple immune cell populations to maintain cutaneous homeostasis, establishing a regulated immune dialogue that limits unnecessary inflammation (Byrd et al., 2018; Belkaid and Segre, 2014). In healthy skin, keratinocytes and immune cells exhibit a highly organized, layer-specific distribution. The epidermis is primarily populated by Langerhans cells and tissue-resident memory T cells (T_RM_), which serve as the first line of immunological surveillance (Hoeffel et al., 2012; Ginhoux et al., 2006). In contrast, the dermis has a more diverse immune landscape. It contains multiple dendritic cell (DC) subsets, including dermal DCs and plasmacytoid DCs (pDCs), as well as T-cell populations such as CD4^+^ helper T-cell subsets (Th1, Th2, and Th17), γδ T cells, and natural killer T (NKT) cells (Zareie et al., 2025). The dermis is further enriched with macrophages, mast cells, neutrophils, innate lymphoid cells (ILCs), mucosal-associated invariant T (MAIT) cells, and fibroblasts, all of which contribute to immune regulation and tissue homeostasis (Nestle et al., 2009; Tong et al., 2015). As shown in Figure 1, these immune cells engage in continuous crosstalk with resident commensal microbes.

Early microbial colonization contributes to the development of anti-infective immunity in infants, indicating that tolerance mechanisms between host and microbiota emerge early in life (Dwyer and Scharschmidt, 2022). For example, commensals such as *S. epidermidis*, *C. acnes*, and *Corynebacterium* species can activate DC–driven regulatory T-cell (Treg) responses, promoting immune tolerance. This is particularly evident during early follicular colonization, when commensal-induced CCL20 expression recruits (Weckel et al., 2023). The enrichment of Tregs in newborns is essential for establishing immune tolerance to skin commensal microbes later in life (Scharschmidt et al., 2015). In addition, tissue-resident MAIT cells develop under the influence of early commensal microbes and become a dominant IL-17–producing effector subset in the skin (Constantinides et al., 2019). γδ T and ILC2 cells also participate in barrier regulation through activation of the IAId–TSLP pathway (Cha et al., 2024).

In adulthood, healthy skin keratinocytes recognize commensals through TLR2, such as lipoteichoic acid(LTA) (Volz et al., 2018) from *S. epidermidis*. This activation induces AMP and IL-10, strengthening the immune barrier. *S. lugdunensis* can also induce LL-37 and CXCL8 expression through the TLR–MyD88 pathway, promoting monocyte and neutrophil recruitment to regulate inflammation and preserve homeostasis (Bitschar et al., 2019). The *S. epidermidis* can further activate keratinocytes through the LTA–TLR2 signaling pathway, thereby inducing the secretion of stem cell factor (SCF) and promoting mast cell recruitment and maturation (Di Nardo et al., 2023). Mature mast cells possess intrinsic antibacterial activity. In addition, under the regulatory influence of dermal fibroblasts, mast cells can develop tolerance toward commensal microbes, which contributes to the maintenance of cutaneous microbial homeostasis (Wang et al., 2017)

Commensal microbial antigens can be presented by DCs to activate CD4^+^T (Th17) and CD8^+^T (Tc17) cells (Naik et al., 2015). These effector cells secrete IL-17A, which stimulates the production of AMPs, enhances epithelial barrier integrity, strengthens antimicrobial defense, and contributes to cutaneous immune homeostasis. In particular, Tc17 cells can acquire CD8^+^ TRM17 which secrete IL-17A (Harrison et al., 2019) and play key crucial role in reinforcing local barrier defense and sustaining long-term immune surveillance in the skin. In parallel, through the presentation of N-formyl methionine–containing peptides(fMet) by non-classical MHC class I molecules(MHC-Ib), commensals such as *S. epidermidis* elicit a distinct population of tissue-resident CD8^+^ T cells endowed with integrated antimicrobial and immunoregulatory functions (Linehan et al., 2018). Unconventional T cells represent an important population of tissue-resident immune cells that continuously contribute to the maintenance of immune balance and barrier homeostasis in the skin. This group primarily includes γδ T cells, MAIT cells, and iNKT cells. Their antigen recognition is restricted by MHC-Ib, enabling them to sense highly conserved microbial metabolites or lipid antigens (Constantinides and Belkaid, 2021).

Notably, *Corynebacterium* can induce the expansion of γδ T cells (Ridaura et al., 2018). MAIT cells are activated via MR1-presented riboflavin metabolites from *S. epidermidis* and fungi, leading to IL-17A production and enhanced mucosal defense (Constantinides et al., 2019).

Although the traditional view held that B cells are largely absent from the skin (Bos et al., 1987), an increasing number of studies have demonstrated that B cells can indeed be detected in the healthy skin of humans and other mammals such as sheep and mice (Geherin et al., 2012; Geherin et al., 2016). Skin B cells include both conventional B-2 cells and innate-like B-1–like cells (Debes and McGettigan, 2019). Most skin B cells are B-2 cells derived from the peripheral blood, which transiently reside in the skin, although their residency duration is not well defined. Innate-like B-1–like cells are more abundant and can generate antibody responses independently of T-cell help, providing rapid humoral protection at the skin barrier. Overall, B cells play essential roles in antibody production, antigen presentation, T-cell activation, and the modulation of inflammatory responses (Debes and McGettigan, 2019).

Recent findings show that immunoglobulins secreted by B-1-like cells can be detected on the skin surface (Baumgarth, 2011; Kearney, JF, 2005). Electron microscopy has revealed that many skin microbes are coated with IgA, IgG, or IgM (Metze et al., 1991; Wilson et al., 2019; Dryla et al., 2005), and secretory IgA can be found in sebaceous glands and follicular ducts (Gebhart et al., 1986; Okada et al., 1988). These evidences indicate that antibodies produced by B cells contribute to the maintenance of skin microbial homeostasis by binding to commensal microbes and modulating their colonization and community stability. In children aged 2 months to 18 years, anti–*S. aureus* IgM appears earliest (Jiang et al., 2015), IgA peaks between ages 4 and 6, and IgG increases steadily with age (Jiang et al., 2015). These observations suggest that the skin, similar to the gut (Macpherson et al., 2018; Grasset et al., 2020; Kelly et al., 2005; Mann and Li, 2014; Kato et al., 2014; Magri et al., 2017), maintains a form of local humoral immune homeostasis in which antibodies cooperate with commensal microbes to provide early protection against pathogens. Early-life colonization by *S. aureus* is capable of eliciting high-titer, antigen-specific antibodies that can persist for years in healthy individuals, indicating potential for long-term immune memory and informing vaccine strategies (Dryla et al., 2005).

A recent study also identified tertiary lymphoid structures (TLOs) in healthy skin (Gribonika et al., 2025). Under stimulation by *S. epidermidis*, Langerhans cells present antigens to CD4^+^ T cells, suppress Foxp3 expression in Tregs, and promote the activation of follicular helper T cells (Tfh) (Gribonika et al., 2025). This process occurs locally in the skin and can extend to systemic immunity. In draining lymph nodes, Tfh cells drive B-cell activation and induce the production of systemic IgG1 and IgG3. In the skin, Tfh cells promote the formation of TLOs, where mucosa-associated antibodies such as IgG2b and IgG2c are generated (Gribonika et al., 2025). These antibodies eventually reach the skin surface to strengthen barrier immunity, while the resulting memory B cells and plasma cells can remain long-term within the tissue. Beyond antibody generation, regulatory B cells (Bregs), which secrete IL-10, constitute an important B-cell subset with immunomodulatory functions (Lykken et al., 2015). Bregs are increasingly recognized for their roles in limiting skin inflammation and maintaining immune balance (Candando et al., 2014). Even under non-inflammatory conditions, Bregs can suppress inappropriate immune activation against commensals, thereby contributing to local immune homeostasis (Geherin et al., 2016). Natural Bregs (nBregs) not only produce antibodies reactive to microbes but may also facilitate the establishment of early skin microbial communities (Gu et al., 2024). The influence of skin microbiota on Bregs requires further investigation and should be a promising area of research.

### 2.2 Barrier integrity and colonization resistance in healthy skin

**Figure 2:**
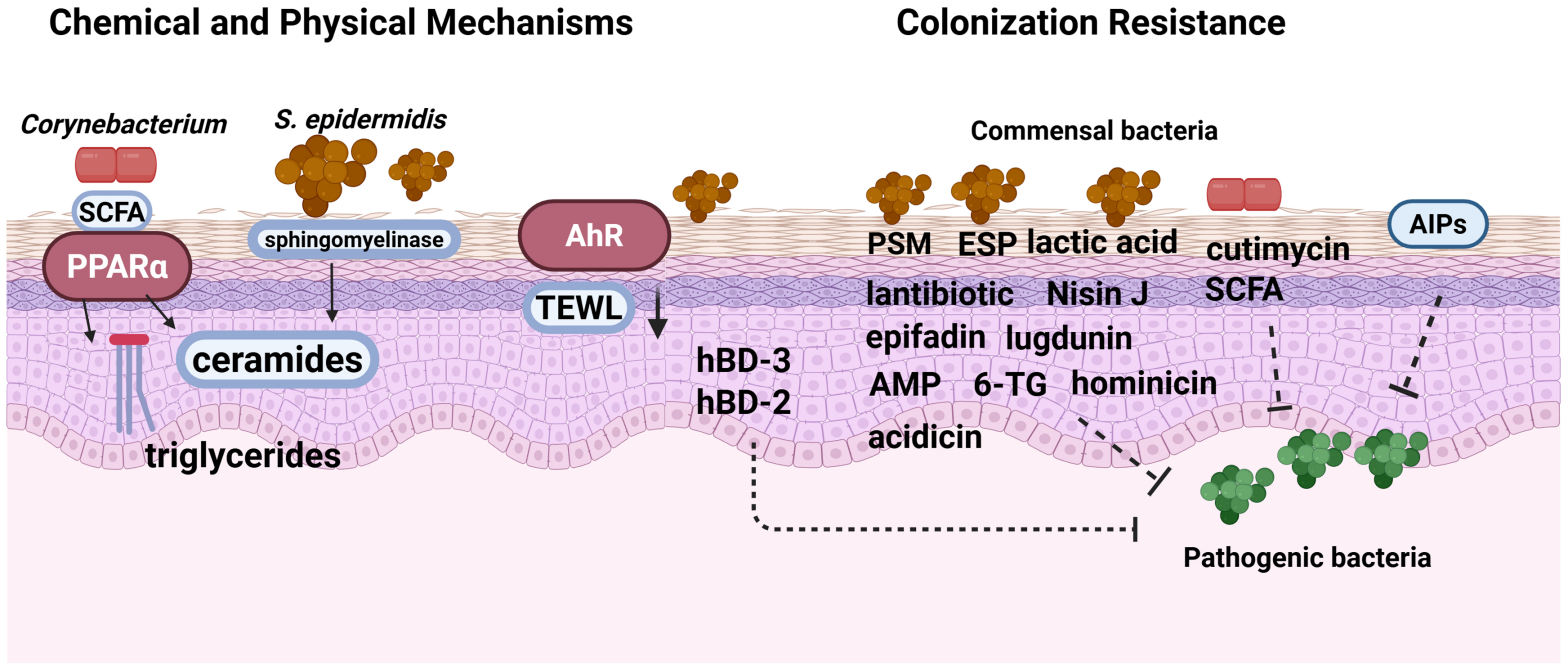
Commensals exert anti-colonization effects against pathogenic microbes. Skin commensals maintain homeostasis through both chemical and physical mechanisms. Commensal skin microbes secrete multiple antimicrobial factors, including PSMs, ESP, lugdunin, lantibiotics, cutimycin, hominicin, 6-TG, AMPs, SCFAs, bacteriocin Nisin J, acidicin and lactic acid, and additionally induce keratinocyte-derived hBDs, all of which contribute to the inhibition of pathogenic bacterial growth. These antimicrobial actions are further regulated through QS pathways, where AIPs from CoNS interfere with *S. aureus* agr signaling to reduce virulence. In addition to antimicrobial and QS-based interactions, commensals support the physical and chemical components of the skin barrier. SCFAs from *C. acnes* activate PPARα signaling to enhance lipid synthesis in keratinocytes. AHR signaling triggered by commensals aids in barrier recovery. *S. epidermidis* also boosts ceramide production via sphingomyelinase secretion, reinforcing the stratum corneum and reducing water loss. Created with BioRender.com. *AIP* autoinducing peptide, *AMP* antimicrobial peptide, *SCFA* short-chain fatty acids, *TEWL* transepidermal water loss, *AHR* aryl hydrocarbon receptor, *PSM* phenol-soluble modulin, *ESP* excretory secretory products, *6-TG* 6-thioguanine, *S. epidermidis Staphylococcus epidermidis.*

Given that the preceding sections primarily address microbiota-driven immune education, other protective mechanisms, such as physical barrier reinforcement, microbial antagonism, and chemical interactions, are summarized in Figure 2. Commensal skin bacteria maintain microbial balance through antimicrobial activity and quorum-sensing (QS)–mediated competition (Glatthardt et al., 2024)

*S. epidermidis* produces a variety of bioactive metabolites, including phenol-soluble modulins (PSMs), extracellular serine protease (ESP), lantibiotic (such as epidermin, Pep5, epilancin K7, epilancin 280, and epilancin A37), epifadin, and lactic acid. These compounds work synergistically to suppress the pathogenicity of *S. aureus* and other opportunistic pathogens (Peschel and Otto, 2013; Salgaonkar et al., 2022; Iwase et al., 2010; Sugimoto et al., 2013; Puls et al., 2024; Torres Salazar et al., 2024; Glatthardt et al., 2020), inhibit biofilm formation, and stimulate keratinocytes to secrete human *β*-defensins 2 and 3 (hBD-2 and hBD-3), thereby enhancing colonization resistance (Rademacher et al., 2019; Lai et al., 2010). Studies have also shown that PSMs can act in synergy with the host-derived antimicrobial peptide LL-37 to further enhance the inhibition of *S. aureus* (Liu et al., 2020).

Other coagulase-negative staphylococci (CoNS) exhibit similar capabilities. For instance, *Staphylococcus lugdunensis* produces lugdunin, *Staphylococcus hominis* produces hominicin, *Staphylococcus chromogenes* produces 6-thioguanine (6-TG), and *Staphylococcus capitis* secretes the broad-spectrum bacteriocin Nisin J. These antimicrobial molecules have demonstrated activity against both *S. aureus* and *C. acnes* (Bitschar et al., 2019; Nakatsuji et al., 2017; Chin et al., 2021; Nguyen et al., 2025; O’Sullivan et al., 2020).

In addition, *C. acnes* can produce short-chain fatty acids (SCFAs), such as succinic acid, butyric acid, and propionic acid, which possess anti-inflammatory and antimicrobial properties that contribute to maintaining skin microbial homeostasis. *C. acnes* also synthesizes cutimycin, a thiopeptide antibiotic that selectively inhibits methicillin-resistant *S. aureus* (MRSA) (Claesen et al., 2020; Shu et al., 2013). Moreover, *Cutibacterium avidum* has recently been shown to produce a novel bacteriocin, acidicin, which exhibits broad-spectrum antimicrobial activity against *C. acnes*, *Lactobacillus*, and *Corynebacterium* species (Koizumi et al., 2023), thereby supporting ecological stability and microbial balance on the skin. QS interference represents another major protective mechanism (Williams et al., 2019). *S. epidermidis* AIP-I and multiple AIPs from *S. hominis* inhibit the *S. aureus* agr system, thereby reducing toxin expression (Otto et al., 2001; Severn et al., 2022).

*C.acnes* contributes to skin homeostasis by secreting SCFAs that activate PPARα signaling, thereby promoting triglyceride and ceramide synthesis in keratinocytes. This process enhances the antimicrobial capacity of the skin barrier, improves water retention, and supports the regulation of skin permeability (Almoughrabie et al., 2023). In addition, the commensal skin microbiota reduces TEWL by modulating AHR signaling and strengthens the skin barrier by promoting tissue repair (Uberoi et al., 2021). *S. epidermidis* further reinforces the physical barrier of the skin through multiple mechanisms. Its secreted sphingomyelinase facilitates ceramide production, which is essential for maintaining the integrity of the stratum corneum (Zheng et al., 2022).

## 3 Disruption and reprogramming of skin microbiota during wound repair

### 3.1 Disruption of the skin microbiome balance

Following skin injury, loss of epidermal barrier integrity exposes the wound bed to resident and environmental microbial populations, reshaping the local microbial landscape. At the same time, the accumulation of wound fluids and the release of nutrients from damaged cells alter the local microenvironment, resulting in changes in temperature, hydration, oxygen availability, and pH compared with intact skin (Kruse et al., 2015). The ecological niche that sustains microbial homeostasis is therefore disrupted, creating conditions that favor the overgrowth of specific microorganisms and leading to microbial dysbiosis within the wound. These microecological alterations interfere with normal host cellular regulatory signaling, causing the dynamic equilibrium characteristic of healthy skin to rapidly collapse following injury.

Although the causal relationship between skin disorders and microbial dysbiosis remains incompletely defined (Byrd et al., 2018; Oh and Voigt, 2025), metagenomic and 16S rRNA sequencing studies consistently demonstrate that wound formation is accompanied by rapid and pronounced reorganization of the skin microbiota (Li et al., 2024; Malone et al., 2017). Relative to intact skin, dominant resident commensal populations decline in abundance, whereas opportunistic pathogens, including *S. aureus*, *Pseudomonas aeruginosa*, *Enterococcus faecalis*, and *Proteus mirabilis*, as well as certain environmental microorganisms, become enriched (Sachdeva et al., 2022). In parallel, the proportion of anaerobic bacteria increases (Coluccio et al., 2024), resulting in wound-associated microbial communities that differ from those observed under homeostatic conditions. In addition to changes in the composition of the community, the wound microbiota exhibit significant functional heterogeneity. Emerging evidence indicates that even classical pathogens such as *S. aureus* can be categorized into “generalist” and “specialist” strains (Kalan et al., 2019; MacLeod, 2019), reflecting distinct ecological adaptations across different microenvironments and host contexts.

Wounds are generally classified into two major types: acute wounds such as burns, blunt trauma and penetrating injuries, and chronic hard-to-heal wounds including diabetic foot ulcers (DFUs), pressure ulcers (PUs), venous leg ulcers (VLUs) and postoperative wounds (Bowler et al., 2001). In acute wounds, dysbiosis of the skin microbiota is often transient and tends to resolve as the epithelial barrier and tissue integrity are restored (Monaco and Lawrence, 2003). Rapid changes in microbial diversity have been associated with wound-healing outcomes, suggesting that dynamic microbial shifts may support resolution of inflammation and tissue regeneration (Loesche et al., 2017). In contrast, chronic or high-risk wounds are characterized by persistent microbial imbalance and prolonged inflammation. This sustained dysbiosis is influenced by host-related factors such as diabetes, neuropathy and vascular dysfunction, as well as microbial factors including excessive pathogen colonization, biofilm formation and interspecies microbial cooperation (Shumba et al., 2019; Ovington, 2003; Xu et al., 2021; Bejarano et al., 1989). When a small number of pathogenic species dominate the wound environment over time, the microbial imbalance can prevent progression through normal healing stages. Therefore, both the type of wound and the host’s capacity to restore microbial homeostasis play crucial roles in determining healing outcomes.

### 3.2 The beneficial roles of commensal microbiota during wound healing

These early changes in the structure of the microbial community do not directly determine the outcome of the healing process but provide the ecological context for subsequent host–microbiota interactions. Within this restructured microbial environment, commensal microorganisms begin to play stage-specific roles that influence the resolution of inflammation, tissue regeneration and barrier restoration.

**Figure 3:**
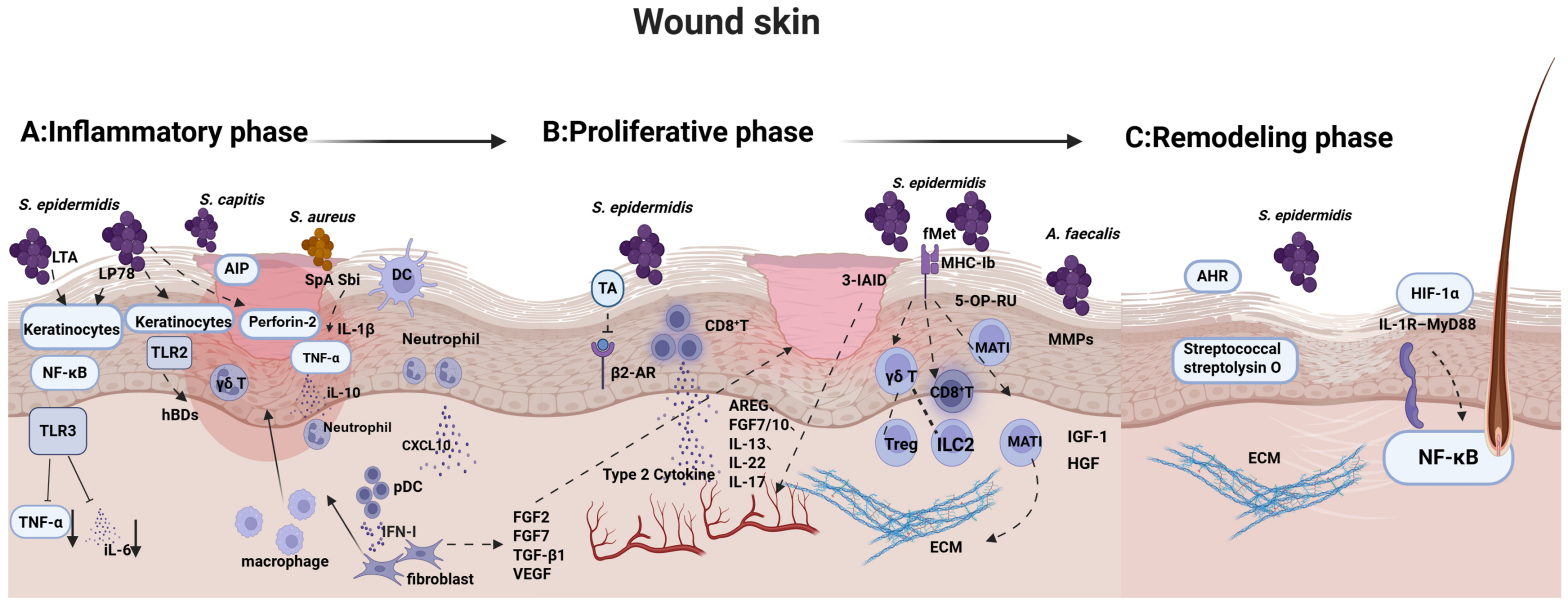
Positive roles of skin microbiota across the major stages of wound healing. **(A)** During the inflammatory phase, *S. epidermidis* modulates keratinocyte NF-κB and TLR signaling through LTA and LP78, limiting excessive inflammation. *Staphylococcus aureus* suppresses inflammatory responses via SpA and Sbi. Concurrently, commensals restrict pathogen overgrowth via QS interference and perforin-2–mediated clearance, and stimulate chemokine and type I interferon signaling to facilitate the transition toward tissue repair. **(B)** In the proliferative phase, re-epithelialization, ECM deposition, and granulation tissue formation are coordinated by reparative immune cells and commensal-derived signals. *S. epidermidis* promotes keratinocyte migration via TA and induces repair-associated unconventional T cells, including γδ T cells, MAIT cells, and tissue-repair CD8^+^ T cells, which produce growth factors and type 2 cytokines. Together with microbial metabolites and host growth factors, these signals accelerate keratinocyte proliferation, fibroblast activation, and neovascularization. **(C)** During the remodeling phase, microbial signals contribute to tissue maturation, barrier restoration, hair follicle regeneration, and scar modulation. Commensal-induced hypoxic signaling and AHR pathway activation promote epidermal reconstruction, whereas specific microbial factors influence collagen reorganization and the outcomes of fibrosis, highlighting the role of the skin microbiota in determining long-term wound architecture and functional recovery. Created with BioRender.com *TA* trace amines, *LP78* lipopeptide 78, *TLR* Toll-like receptor, *TNF* tumor necrosis factor, *DC* dendritic cell, *pDC* plasmacytoid dendritic cell, *MHC* major histocompatibility complex, *MMPs* matrix metalloproteinases, *AHR* aryl hydrocarbon receptor, *ECM* extracellular matrix, *LTA* lipoteichoic acid, *HIF-1α* hypoxia-inducible factor 1 alpha, *β2-AR* beta-2 adrenergic receptor, *3-IAID* indole-3-aldehyde, *fMet* N-formylmethionine, *S. epidermidis* *Staphylococcus epidermidis*, *S. aureus Staphylococcus aureus*, *S. capitis Staphylococcus capitis*, *A.faecalis Alcaligenes faecalis.*

This section focuses on how skin microbiota dynamically regulate different stages of wound healing, including inflammation, proliferation, and remodeling, highlighting the gradual shift from instability to recovery which is shown in Figure 3. This process involves not only changes in microbial community structure but also bidirectional regulation by the host immune environment, forming a complex and dynamic host–microbe interaction network that ultimately determines wound healing outcomes. It is worth noting that the phases of wound healing often overlap, and commensal microbes may play a role across multiple stages. The discussion here is organized according to the stage at which they play a predominant role.

#### 3.2.1 Inflammatory phase

During the inflammatory phase of wound healing, the tissue is characterized by the recruitment and activation of innate immune cells, including keratinocytes, macrophages, and neutrophils (Peña and Martin, 2024). These cells originate not only from local tissues but also migrate into the wound site through vascular extravasation. Immune regulation at this stage involves not only host cells but also commensal microorganisms, which help maintain inflammatory balance and limit excessive immune responses.

In controlling excessive inflammation, *S. epidermidis* secretes LTA, which mildly activates the NF-κB signaling pathway in keratinocytes while suppressing TLR3-mediated overexpression of pro-inflammatory cytokines such as TNF-*α* and IL-6, thereby reducing inflammatory intensity (Lai et al., 2009). In addition, the lipopeptide LP78 produced by *S. epidermidis* activates the *β*-catenin signaling pathway, further inhibiting TLR3-NK-κB inflammation (Li et al., 2019).

Regarding infection control, *Staphylococcus capitis* secretes AIPs that antagonize the agr signaling system of MRSA, thereby alleviating pathogen-induced skin pathology (Paharik et al., 2017). *S. epidermidis* can also induce the expression of perforin-2 in epithelial cells and γδ T cells, enhancing the clearance of *S. aureus* (Pastar et al., 2021; Strbo et al., 2019; Pastar et al., 2020). At the same time, it stimulates keratinocytes to produce antimicrobial peptides such as hBDs by activating TLR2 signaling, thereby further strengthening local antimicrobial defense (Lai et al., 2010)

In immune cell recruitment, commensal bacteria can stimulate neutrophils to release the chemokine CXCL10, which subsequently attracts plasmacytoid dendritic cells (pDCs) and promotes the production of type I interferons (IFN-I) (Di Domizio et al., 2020). IFN-I then activates macrophages and fibroblasts to express various tissue growth factors, including FGF2, FGF7, TGF-β1, and VEGF, facilitating the transition from the inflammatory phase to the proliferative phase (Di Domizio et al., 2020).

Notably, *S. aureus* can also exert pro-healing effects under certain conditions. Its surface proteins, staphylococcal protein A (SpA) and staphylococcal binder of immunoglobulin (Sbi) induce the production of IL-1β and TNF-*α*, while elevated IL-10 levels in the wound promote neutrophil recruitment, thereby restricting bacterial dissemination to superficial layers (Gonzalez et al., 2019). This creates a “controlled inflammation” environment that supports the progression of wound healing.

#### 3.2.2 Proliferative phase

The proliferative phase is characterized by several key processes, including angiogenesis, fibroblast recruitment and proliferation, ECM deposition, and re-epithelialization (Peña and Martin, 2024). During this stage, inflammatory cells, particularly macrophages polarized toward an M2 phenotype, act as “orchestrators” of tissue repair by coordinating multiple cell types to construct granulation tissue that replaces the damaged dermal structure.

*S. epidermidis* contributes to re-epithelialization by producing trace amines (TAs) through the enzyme SadA. These metabolites suppress the activity of β2-adrenergic receptors (β2-AR) on keratinocytes, thereby releasing the receptor-mediated inhibition of cell migration and accelerating keratinocyte motility (Luqman et al., 2020).

Unconventional T cells also participate in repair during this stage. Resident γδ T cells secrete IGF-1 to directly promote epithelial regeneration and further support tissue homeostasis by recruiting Tregs and ILC2s (Constantinides and Belkaid, 2021). Skin MAIT cells induced by 5-OP-RU from *S. epidermidis* upregulate genes related to angiogenesis and tissue repair, including angiopoietins, IGF-1, and HGF (Constantinides et al., 2019). Notably, MAIT cells reside just beneath the basement membrane, which serves as a structural scaffold for progenitor cell migration and adhesion during wound healing.

*S. epidermidis* promotes a specialized subset of CD8^+^ T cells via non-classical H2-M3–restricted MHC-Ib antigen presentation. On the other hand, antigens derived from *S. epidermidis* can also be presented through classical MHC class I molecules to generate commensal-specific CD8^+^ T cells. Importantly, these classical MHC-restricted CD8^+^ T cells exhibit a pronounced tissue-repair phenotype and produce IL-13 together with other type 2–associated cytokines. They also secrete growth factors such as AREG, FGF7, and FGF10. In addition, these cells can express IL-22 and under inflammatory or tissue-damage conditions IL-17. This functional plasticity enables them to coordinate antimicrobial defense with tissue repair (Harrison et al., 2019; Naik et al., 2015; Linehan et al., 2018). In addition, tissue growth factors produced during the preceding inflammatory phase which is mentioned before such as FGF2, FGF7, TGF-β1, and VEGF, continue to play essential roles in the proliferative phase, promoting angiogenesis and tissue remodeling (Di Domizio et al., 2020). Moreover, the evidence also indicates that the microbial metabolite indole-3-aldehyde (3-IAld) enhances neovascularization within the wound microenvironment (Ma et al., 2022). Together, these factors promote keratinocyte proliferation, fibroblast activation, ECM remodeling, and angiogenesis to accelerate wound healing.

Importantly, studies have shown that non-typical skin commensals may also contribute to repair during the proliferative phase. For example, *Alcaligenes faecalis* secretes peptides that modulate keratinocyte MMPs expression, enhance re-epithelialization, and promote cell migration and proliferation, indicating that a broader array of skin microbes may possess pro-healing potential (White et al., 2024).

#### 3.2.3 Remodeling phase

The remodeling phase is characterized by the gradual replacement of granulation tissue with scar tissue, which contains fewer cells and displays a more organized structure. This stage marks the final phase of wound healing, during which cellular migration, inflammatory responses, angiogenesis, and matrix deposition that were active in earlier phases progressively subside, leading to structural stabilization of the tissue (Peña and Martin, 2024). As collagen fibers undergo reorganization and crosslinking, the mechanical strength of the tissue increases, ultimately completing the long-term repair of the wound.

During this period, hair follicles begin to regenerate. Commensal bacteria can induce a mild hypoxic microenvironment that activates hypoxia-inducible factor 1α (HIF-1α) in keratinocytes, thereby enhancing hair follicle regeneration. Specifically, this process drives glutamine metabolism and the expression of interleukin-1β (IL-1β). IL-1β activates the NF-κB signaling pathway through the IL-1 receptor–MyD88–dependent axis, working together with HIF-1α to promote hair follicle regeneration, epidermal reconstruction, and re-epithelialization (Wang et al., 2021, 2023).

In addition, when AHR signaling is attenuated, subsequent activation by AHR ligands may enhance barrier recovery during the late stages of healing (Uberoi et al., 2021). Notably, certain skin microbes can directly modulate scar formation. For example, Streptococcal streptolysin O has been shown to reduce the development of hypertrophic scars and keloids, suggesting that microbiota influence not only regeneration but also the extent of fibrosis during the remodeling phase (Tomic-Canic et al., 2007)

In addition, B cells and Bregs have emerged as important regulators of inflammation resolution and tissue repair during wound healing (Diehl et al., 2025; Sîrbulescu et al., 2017; Oliveira et al., 2010; Nishio et al., 2009; Yin and Wu, 2025). However, the mechanisms by which the skin microbiota orchestrates beneficial B-cell responses within the wound microenvironment remain poorly defined. Understanding how the microbiota modulates B cell responses may provide insights relevant to wound regeneration.

## 4 Restoration of skin microbiota homeostasis after wound closure

Following wound closure, the skin does not simply revert to its pre-injury homeostatic state. Although re-epithelialization and tissue reconstruction restore barrier continuity, the post-wound skin remains distinct from unwounded skin at both structural and immunological levels. Remodeling of the dermal matrix, altered vascular and neural architecture, and changes in skin appendages reshape the local microenvironment, thereby influencing microbial recolonization dynamics.

In parallel, wound-induced immune remodeling persists after closure and contributes to the establishment of a post-injury steady state. CD4^+^T_RM_ and CD8^+^ T_RM_ cells during tissue damage are maintained locally and support rapid immune responses upon antigen re-encounter (Mueller and Mackay, 2016; Wilk and Mills, 2018; Iijima and Iwasaki, 2015). Tissue-resident and circulating memory B cells further support protective immunity through local persistence and antibody-mediated defense (Palm and Henry, 2019). These immune adaptations reset local immune thresholds and shape a post-wound immune landscape that differs from the original homeostatic state.

Within this remodeled tissue and immune context, restoration of skin microbiota homeostasis reflects a functional rather than compositional recovery. A healthy post-wound skin microbiome is characterized by regained microbial diversity, structural stability, and effective colonization resistance, accompanied by immune tolerance that limits unnecessary inflammation as mentioned above. Failure to establish this coordinated microbiota–immune equilibrium predisposes healed wounds to instability. In such cases, reduced microbial diversity, persistent dominance of opportunistic taxa, and sustained immune activation render the skin highly sensitive to external stimuli. Host factors such as diabetes, vascular dysfunction, neuropathy, and immune senescence may further impair long-term homeostasis recovery through endogenous biological drivers, thereby increasing the risk of wound recurrence (Berlanga-Acosta et al., 2023).

## 5 Microbiota-based strategies and safety considerations in wound repair

Excessive or non-selective antimicrobial interventions may disrupt the skin’s microbial balance, potentially impairing the beneficial roles of commensals in immune regulation and wound repair (Davies and Davies, 2010). Therefore, microbiota-based therapeutic approaches that aim to restore or preserve microbial homeostasis represent a promising strategy to support wound healing while minimizing collateral damage to the native skin microbiota.

Microbiota-targeted therapeutic strategies for skin disease can generally be classified into three major categories. The first approach employs live microorganisms as therapeutic agents, including the application of the probiotics, commensal microbiome, genetically engineered bacteria, or interventions that promote the growth of beneficial microorganisms (Lopes et al., 2017; Valdez et al., 2005; Peral et al., 2009). This category also encompasses microbial transplantation techniques (Nezhadi et al., 2024), such as skin microbiota transfer, aimed at replacing dysbiotic microbial communities with stable and diverse populations. The second strategy focuses on selectively eliminating pathogenic or dysregulated microbes using antibiotics or bacteriophage-based interventions. In particular, targeted bacteriophage approaches can attenuate bacterial virulence, thereby promoting wound healing (Cao et al., 2019; Wang et al., 2024). The third approach focuses on microbiota-derived bioactive molecules, such as bacterial metabolites, which can be utilized as therapeutic agents to modulate host physiology and immune responses (Di Domizio et al., 2020; Severn and Horswill, 2023; Takahashi et al., 2021).

Most current bacterial therapies rely on non–skin-derived probiotics (Kadwaikar and Shinde, 2025), such as *Lactobacillus plantarum* and *Lactobacillus reuteri* (Peral et al., 2009, 2010; Khodaii et al., 2019), Recently, postbiotics have also emerged as promising agents for promoting wound healing (Hashemi et al., 2025). In contrast, the direct application of skin commensals in wound treatment remains relatively uncommon. This disparity may be attributed to the complexity and dynamic nature of the wound microenvironment, as well as safety concerns regarding potential opportunistic behavior of commensals under dysregulated immune conditions. Although conventional probiotic-based therapies are relatively well established, utilizing skin commensals or their metabolites to promote wound healing represents a promising yet underexplored strategy for wound management.

In contrast, the therapeutic application of skin commensals in other skin conditions, such as atopic dermatitis and acne, has advanced substantially and has even entered clinical trial stages. Table 1 summarizes existing examples of skin commensal–based therapies in dermatological diseases. Similar to these conditions, wounds also require appropriately activated immune responses and antimicrobial factor production to prevent pathogenic overgrowth. Therefore, therapeutic experiences from other skin disorders offer valuable insights for the development of skin commensal–based approaches in wound healing.

**Table 1:** Summarizes representative examples of current applications of skin microbiota in cutaneous interventions.

Skin type	Intervention type	Skin bacteria application	Preclinical/clinical trial phase	Sample	Observed effect	Reference
Wound	Bacterial metabolites	*S. epidermidis*	Preclinical phase	Mice	Trace amines produced by skin microbiota promote wound repair.	Luqman et al. (2020)
Wound	Microbiota-induced immune activation	*S. epidermidis*	Preclinical phase	Mice	Accelerate wound healing by initiating a type I IFN–mediated innate response through neutrophil–pDC–macrophage signaling, promoting early inflammation and growth factor–driven tissue repair.	Di Domizio et al. (2020)
Wound	Microbiota-induced immune activation	*S. epidermidis*	Preclinical phase	Mice	Commensal-specific TC17 cells facilitate tissue repair by rapidly adapting to injury via their poised type 2 immune potential.	Harrison et al. (2019)
AD	Vaccination (bacterial extract)	*C. acnes*	Preclinical phase	Mice	Induction of regulatory T cells and Th1 immune responses	Kitagawa et al. (2011)
AD	Live bacteria	*S. cohnii*	Preclinical phase	Mice	Suppressed dermatitis and type 2 cytokines	Ito et al. (2021)
AD	Live bacteria	*R. mucosa*	Clinical Trial phase	10 adults, 5 children	≥50% reduction in SCORAD; improved CDLQI and FDLQI in children	Myles et al. (2018)
AD	Live bacteria	*S. hominis*	Preclinical phase	Pig and Mice	Antibacterial activity against *S. aureus*	Nakatsuji et al. (2017)
AD	Live bacteria	*S. hominis*	Clinical Trial phase	5 adults	Increased anti-*S. aureus* activity	Nakatsuji et al. (2017)
AD	Live bacteria	*S. hominis A9*	Preclinical phase	Mice	Reduced erythema, TEWL, disease score, and QS inhibition	Nakatsuji et al. (2021)
AD	Live bacteria	*S. hominis A9*	Clinical Trial phase	54 adults	Reduced *S. aureus* load, increased Sh A9 DNA, no clinical change	Nakatsuji et al. (2021)
AD	Live bacteria	*R. mucosa*	Clinical Trial phase	15 children	Improved skin barrier and reduced steroid use; no severe AEs	Myles et al. (2020)
Psoriasis	Live bacteria	*S. cohnii*	Preclinical phase	Mice	Suppressed inflammatory phenotype	Ito et al. (2021)
Acne vulgaris	Engineered Live bacteria	*C. acnes*	Preclinical phase	≥2 Mice/group	Regulated sebaceous lipid secretion via NGAL	Knödlseder et al. (2024)
Acne vulgaris	Engineered Live bacteria	*S. epidermidis*	Preclinical phase	5 Mice/group	*S. epidermidis*-generated electricity inhibited *C. acnes* growth	Marito et al. (2021)
Melanoma	Engineered Live bacteria	*S. epidermidis*	Preclinical phase	8 Mice/group	T-cell mediated suppression of local and metastatic melanoma	Chen et al. (2023)

AD, atopic dermatitis; *C. acnes, Cutibacterium acnes; R. mucosa, Roseomonas mucosa; S. hominis, Staphylococcus hominis; S. cohnii, Staphylococcus cohnii; S. epidermidis, Staphylococcus epidermidis; S. aureus, Staphylococcus aureus*; SCORAD, Scoring Atopic Dermatitis; CDLQI, Children’s Dermatology Life Quality Index; FDLQI, Family Dermatology Life Quality Index; TEWL, transepidermal water loss; QS, quorum sensing; NGAL, neutrophil gelatinase-associated lipocalin.

As summarized in Table 1, when appropriately selected and precisely regulated, the human microbiota can minimize potential pathogenic risks while amplifying beneficial biological functions, thereby demonstrating considerable potential as a therapeutic strategy for skin wound healing. Based on the positive roles of microbes observed during the wound repair process, three potential application directions are further proposed.

(1) Utilization microbial-derived molecules: Further studies are needed to isolate and characterize immunomodulatory molecules secreted by skin commensals. During the inflammatory phase, microbial factors such as LTA, LP78, AIPs, perforin-2 from *S. epidermidis*, along with host-derived human *β*-defensins induced by *S. epidermidis*, as well as SpA and Sbi from *S. aureus*, may contribute to inflammation resolution and immune cell recruitment. In the proliferative phase, commensal-derived metabolites and peptides, including fMet, 5-OP-RU, 3-IAId and TA, may promote keratinocyte proliferation and re-epithelialization. During the remodeling phase, bacterial products such as streptolysin O from Streptococcus species may influence extracellular matrix remodeling and tissue maturation. Collectively, these microbe-derived molecules may act in a coordinated, stage-dependent manner to support efficient wound healing.
  In addition, the aforementioned bioactive molecules produced by skin commensals, including PSMs, ESP, epilancin A37, epifadin, lactic acid, lugdunin, synergistic AMPs, 6-thioguanine (6 TG), cutimycin, short-chain fatty acids (SCFAs), and autoinducing peptides (AIPs), collectively contribute to antimicrobial and anti-colonization effects through complementary mechanisms. These molecules act by directly inhibiting pathogen growth, interfering with quorum sensing dependent virulence systems, disrupting biofilm formation, and enhancing colonization resistance. While their stabilizing roles have been demonstrated primarily in the context of healthy skin, it is highly likely that they also contribute to wound healing by limiting pathogen spread and reducing infection risk, which warrants further investigation.
(2) Strain Engineering: In addition to isolating beneficial microbe-derived molecules, synthetic biology approaches can be employed to engineer commensal strains by selectively deleting antibiotic resistance genes and virulence factors while enhancing the expression of beneficial molecules, thereby improving both safety and functional specificity. Engineered commensals may exert therapeutic effects not only through the secretion of bioactive factors but also by directly modulating host immune responses, such as stimulating neutrophils or CD8^+^ T cells and activating key signaling pathways including NF-κB, HIF-1α and the AHR signal. Furthermore, the integration of controllable-release biomaterial delivery systems may enable precise spatial and temporal regulation of engineered microbes, limiting excessive bacterial proliferation and maintaining microbial homeostasis within the wound microenvironment.
(3) B cell–targeted therapeutic strategies and pre-immune conditioning: Potential therapeutic approaches targeting B cells may involve the selective depletion of pathogenic B-cell subsets while preserving or enhancing Bregs, thereby enabling a more precise immune–microbiota–coordinated modulation strategy to restore cutaneous immune homeostasis. Beyond therapeutic intervention after injury, the concept of pre-immune conditioning, in which prevention precedes treatment, warrants further exploration. For high-risk populations, such as patients with diabetes or elderly individuals prone to pressure ulcers, commensal-based strategies could be applied under healthy skin conditions to pre-activate TLOs and induce antigen-specific antibody responses by using vaccine-like effect. Such immune priming may allow a more rapid and efficient initiation of wound repair processes following tissue injury, ultimately improving healing outcomes and reducing the risk of chronic wound development.

Despite their therapeutic potential, the application of microbes in wound healing faces several critical challenges. Rigorous risk assessment is essential, given the dual roles of commensal bacteria and the heterogeneity of wound types. For commensal strains, it is important to recognize that commensal bacteria can sometimes act as opportunistic pathogens, particularly in immunocompromised individuals (Brown and Horswill, 2020; Ziebuhr et al., 2006). Therefore, key risk factors—including their infectious potential in vulnerable hosts (Otto, 2009; Vuong and Otto, 2002), intrinsic antibiotic resistance, Fišarová et al. (2021), Meric et al. (2015), Dinić et al. (2024), virulence gene carriage (Zhou et al., 2020; Cau et al., 2021), and biofilm-forming potential (Otto, 2008) must be thoroughly characterized. Identifying the most suitable strains and potentially engineering them for enhanced safety and functionality represents a promising direction.

In addition, wound characteristics exhibit substantial heterogeneity, including distinctions between acute and chronic wounds, different stages of the healing process, and considerable inter-individual variation. Without appropriate patient and wound stratification, microbiota-based interventions may carry significant risks, such as sepsis (Joubert et al., 2022). Therefore, key factors including infection status, wound size, and healing phase should be carefully assessed prior to the administration of microbial therapies.

Another major barrier lies in regulation. Existing frameworks were designed primarily for small-molecule drugs and are not fully equipped to ensure the safety, quality control, and long-term monitoring of live biotherapeutic products. These challenges underscore the need for context-specific safety frameworks, refined microbial screening strategies, and updated regulatory pathways to support the safe and effective clinical translation of microbiota-based wound therapies.

## 6 Conclusion and perspectives

In this review, we summarized current evidence on the dynamic involvement of the skin microbiota across healthy homeostasis, injury-induced disruption, and subsequent wound repair, with a particular focus on its roles in maintaining barrier integrity and supporting tissue regeneration following damage. Increasing evidence indicates that both microbial communities and microbe-derived metabolites actively participate in host–microbiota communication during wound healing, shaping immune responses and local tissue remodeling.

Looking forward, achieving greater precision in microbiota-based approaches to wound repair will require deeper mechanistic understanding of how microbial signals interact with host tissues. Future research should prioritize the identification of key microbe-derived metabolites and structural components, clarification of their host signaling pathways, and definition of their spatiotemporal effects across different stages of wound healing, with particular attention to their modulation of immune cells such as T cells, B cells, and macrophages. Moreover, considering that wound environments often harbor diverse microbial communities, future studies should also explore interspecies interactions among skin-resident microbes. Most current research remains limited to simplified dual-species models, which fail to capture the complexity of multiple microbial communities commonly found in wounds. Therefore, developing advanced *in vitro* and *in vivo* systems that reflect the dynamics of multi-species microbial communities will be essential. Importantly, beyond wound closure, the establishment of a stable post-healing homeostatic state and the prevention of its subsequent disruption deserve greater attention. Macroscopic wound closure should not be regarded as the therapeutic endpoint; rather, restoration of immune barrier function and effective colonization resistance are critical hallmarks of true skin health recovery. Advancing this knowledge will be essential for developing microbiome-based strategies.
